# Non-Pharmacologic Manual Therapies for Postoperative Bowel Dysfunction: A Systematic Review and Meta-Analysis

**DOI:** 10.3390/jcm15135245

**Published:** 2026-07-04

**Authors:** Alexander Ponce, Emily R. Stack, Oliver Perrine, Casey Hawes

**Affiliations:** 1 College of Osteopathic Medicine, William Carey University, 710 William Carey Parkway, Hattiesburg, MS 39401, USA; 2 Lakeland Surgical Clinic, 971 Lakeland Drive, Suite 1460, Jackson, MS 39216, USA

**Keywords:** postoperative bowel dysfunction, postoperative ileus, osteopathic manipulative treatment, abdominal massage, manual medicine, postoperative recovery, gastrointestinal motility, OMT, OMM

## Abstract

**Background**: Postoperative bowel dysfunction, including delayed gastrointestinal recovery and postoperative ileus, is a common complication that increases morbidity and prolongs hospitalization. In this systematic review and meta-analysis, we evaluated the effects of manual therapies on postoperative bowel function. **Methods**: MEDLINE/PubMed, Google Scholar, the Cochrane Library, Semantic Scholar, and ClinicalTrials.gov were searched from database inception up to 17 March 2026, and studies evaluating osteopathic manipulative treatment (OMT) or abdominal massage in postoperative patients were included in our analysis. Risk of bias and certainty were assessed using validated study design-specific tools, including the Grading of Recommendations Assessment, Development, and Evaluation (GRADE) framework. Random-effects meta-analyses were performed for the prespecified outcomes of time to first bowel movement, time to first flatus, and hospital length of stay. **Results**: Seventeen studies met our inclusion criteria. Both OMT and abdominal massage were associated with a significantly shorter time to first bowel movement compared with controls (OMT: mean difference [MD] −0.57 days, 95% CI −0.96 to −0.18; abdominal massage: MD −0.91 days, 95% CI −1.47 to −0.35). OMT was also associated with reduced hospital length of stay (MD −2.46 days, 95% CI −4.52 to −0.41), while time to first flatus demonstrated favorable but non-significant trends, with substantial heterogeneity. **Conclusions**: Manual therapy may be associated with earlier postoperative bowel recovery, although heterogeneity and methodological limitations warrant cautious interpretation. Further high-quality multicenter studies are needed to clarify the clinical significance and reproducibility of these findings.

## 1. Introduction

Postoperative bowel dysfunction, encompassing a wide range of gastrointestinal symptoms, including but not limited to delayed passage of flatus, abdominal distention, nausea, and vomiting, is a common complication following surgery and is associated with increased patient discomfort, delayed recovery, prolonged hospital stay, and increased healthcare costs [1,2]. Postoperative ileus, a severe form of postoperative bowel dysfunction that occurs in as many as 10–30% of patients undergoing abdominal surgery, is defined as a temporary impairment of gastrointestinal motility after surgery that is not associated with a mechanical obstruction [1,3]. The development of postoperative bowel dysfunction is multifactorial and may result from the effects of anesthesia, surgical stress response, inflammation, opioid analgesics, and postoperative mobility—all factors that may contribute to a delay in gastrointestinal motility and recovery [2,3]. As the return of bowel function is often a key milestone for hospital discharge, delays in gastrointestinal recovery can significantly impact postoperative recovery and healthcare utilization [1,2].

Current management of postoperative bowel dysfunction primarily focuses on pharmacologic interventions and supportive care [4,5,6]. Opioid-sparing pain management strategies are commonly used to reduce opioid-induced gastrointestinal hypomotility and subsequent delays in postoperative bowel function [4], while pharmacologic agents such as stool softeners, stimulant laxatives, osmotic agents, and prokinetic agents are frequently used to promote bowel motility and reduce constipation in the postoperative period, although evidence supporting routine use varies [5]. Peripherally acting mu-opioid receptor antagonists, such as alvimopan, have been shown to accelerate gastrointestinal recovery and reduce length of hospital stay following bowel surgery by counteracting opioid-induced gut hypomotility without affecting analgesia [6]. Despite these interventions, pharmacologic management is not always effective and may be associated with side effects, increased cost, and variability in patient response, leading to continued interest in adjunctive therapies to improve postoperative bowel function and recovery [4,6].

Non-pharmacologic strategies are also commonly used to promote the return of bowel function following surgery [7,8,9]. Early ambulation, early enteral feeding, and adequate hydration are key components of Enhanced Recovery After Surgery (ERAS) protocols and have been shown to reduce postoperative complications and shorten length of hospital stay by promoting an earlier return of gastrointestinal function [7]. Chewing gum, a form of sham feeding, has also been studied as a simple intervention to stimulate gastrointestinal motility through activation of the cephalic–vagal reflex and increased gastrointestinal hormone secretion [8,9]. Randomized controlled trials and meta-analyses testing sham feeding suggest reductions in time to first flatus and time to first bowel movement following surgery, although the magnitude of benefit varies between studies [8,9]. Despite the use of these non-pharmacologic strategies, postoperative bowel dysfunction remains a common postoperative complication, suggesting that additional adjunctive therapies may be beneficial to further improve gastrointestinal recovery [7,9].

Manual therapy, including osteopathic manipulative treatment (OMT) and abdominal massage, has been investigated as a potential adjunctive therapy to improve postoperative bowel function [10,11,12,13]. Several physiological mechanisms have been hypothesized, including modulation of autonomic nervous system activity, reduction in sympathetic tone, enhancement of parasympathetic activity, improvement in lymphatic and venous return, and mechanical stimulation of the gastrointestinal tract [10].

OMT may influence gastrointestinal recovery through effects on autonomic regulation and circulatory function, whereas abdominal massage may facilitate gastrointestinal transit through direct mechanical stimulation of the abdominal wall and underlying viscera [10,11,12,13]. However, much of the mechanistic rationale is based on physiologic theory and indirect evidence, and the extent to which these mechanisms contribute to postoperative gastrointestinal recovery remains incompletely understood.

Clinical evidence evaluating these interventions in postoperative patients has yielded mixed results. Studies of OMT have reported improvements in outcomes such as time to first flatus, time to first bowel movement, postoperative ileus, and hospital length of stay, although findings have not been consistent across investigations [10,11,12]. Similarly, abdominal massage has been evaluated as a non-invasive intervention to support gastrointestinal recovery, with some studies demonstrating earlier return of bowel function and others reporting limited or no benefit [13]. Given the heterogeneity in study designs, patient populations, and treatment protocols, the overall effectiveness of manual medicine in the management of postoperative bowel dysfunction remains uncertain and warrants further evaluation.

Therefore, the objective of this systematic review is to evaluate the effect of manual therapy, including OMT and abdominal massage, compared with standard postoperative care or no manual therapy on the recovery of bowel function and the prevention and treatment of postoperative bowel dysfunction in patients undergoing surgery.

## 2. Materials and Methods

### 2.1. Protocol

This systematic review was conducted in accordance with the Preferred Reporting Items for Systematic Reviews and Meta-Analyses (PRISMA) 2020 reporting guidelines [14]. Although this review was not prospectively registered, eligibility criteria, outcomes of interest, search strategies, study selection procedures, and planned analytical methods were established prior to study screening and data extraction. The completed PRISMA 2020 Checklist and PRISMA 2020 for Abstracts Checklist are provided in the Supplementary Files S1 and S2.

### 2.2. Eligibility Criteria

The review question was structured according to the Population, Intervention, Comparator, Outcome, and Study Design (PICOS) framework. The population included patients experiencing postoperative bowel dysfunction or undergoing postoperative recovery where gastrointestinal function outcomes were reported. The interventions of interest were hands-on manual medicine therapies, including OMT and abdominal massage. Comparators included standard postoperative care, sham treatment, placebo interventions, or no manual therapy. No single primary outcome was prespecified for this review. Given the heterogeneity of the available literature, the review evaluated multiple clinically relevant indicators of postoperative gastrointestinal recovery. Outcomes selected for quantitative synthesis included time to first bowel movement, time to first flatus, and hospital length of stay. Secondary outcomes included postoperative ileus incidence, bowel sounds, dietary tolerance, constipation-related outcomes, and other measures of gastrointestinal recovery. Quantitative synthesis was performed when sufficient data were available. Eligible study designs included randomized controlled trials, non-randomized interventional studies, cohort studies, and other clinical studies reporting human patient-level outcomes.

Studies were eligible for inclusion if they involved patients undergoing postoperative recovery in whom gastrointestinal recovery outcomes were assessed, evaluated a form of hands-on manual therapy as an intervention, and reported human patient-level clinical or physiological outcomes. For the purposes of this review, manual therapy was defined as direct therapeutic intervention administered manually by a practitioner without the use of mechanical devices, instruments, or needle-based modalities. All clinical studies evaluating such manual therapies for the management of postoperative bowel dysfunction were eligible for inclusion regardless of study design, clinical context, or outcome measure. Narrative reviews, systematic reviews, studies evaluating pharmacologic interventions, and studies assessing interventions delivered primarily through devices or instruments (e.g., acupuncture needles or electrically powered massage devices) were excluded.

### 2.3. Information Sources and Search Strategy

A comprehensive literature search was conducted to identify clinical studies evaluating the use of manual medicine in patients with postoperative bowel dysfunction. The databases MEDLINE via PubMed (National Library of Medicine, Bethesda, MD, USA), Google Scholar (Google, Mountain View, CA, USA), the Cochrane Library (Wiley, Hoboken, NJ, USA), Semantic Scholar (Allen Institute for AI, Seattle, WA, USA), and ClinicalTrials.gov (National Library of Medicine, Bethesda, MD, USA) were searched from database inception to 17 March 2026. Only studies published in English were considered for inclusion. Search strategies were adapted for each database to account for differences in indexing systems, controlled vocabulary, and search functionality, while maintaining consistent conceptual domains related to postoperative bowel dysfunction and manual medicine. Detailed database-specific search strategies are provided in Appendix A.

Search terms included combinations of keywords and controlled vocabulary related to manual medicine and postoperative bowel dysfunction, such as “postoperative”, “postoperative ileus”, “paralytic ileus”, “massage therapy”, and “osteopathic manipulative treatment.” In MEDLINE/PubMed, Medical Subject Headings (MeSH) were used to optimize sensitivity. Google Scholar searches prioritized articles with high relevance to the topic by limiting queries to titles. The Cochrane Library and Semantic Scholar were searched using comparable keyword combinations to ensure comprehensive retrieval of relevant studies. ClinicalTrials.gov utilized the name of the condition and manual therapy in the other terms section.

### 2.4. Study Selection and Data Extraction

**Figure 1:**
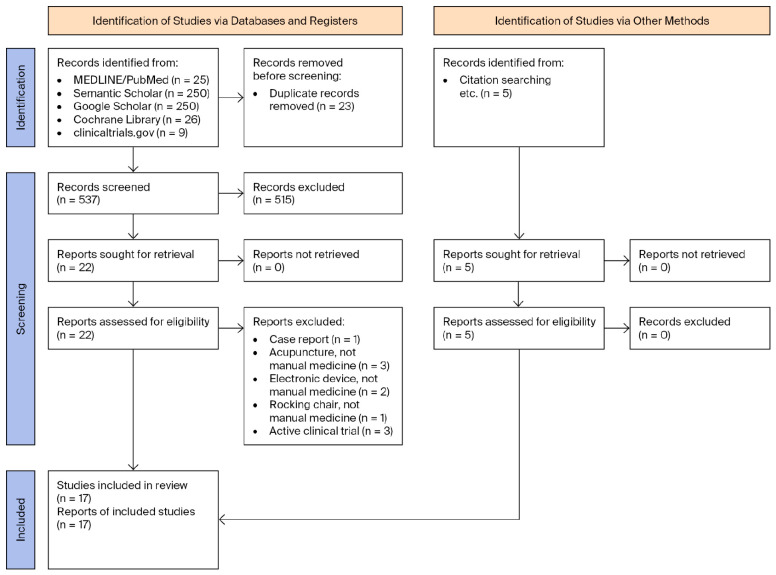
PRISMA 2020 flow diagram of study selection process. PRISMA: Preferred Reporting Items for Systematic Reviews and Meta-Analyses.

Literature searches, title and abstract screening, full-text eligibility assessment, and data extraction were performed by two authors (A.P. and O.P.) using predefined eligibility criteria. Screening and study selection were conducted independently, and any discrepancies between reviewers were resolved through discussion and consensus. Reasons for exclusion at the full-text stage are detailed in Figure 1 [14]. Risk of bias assessments were applied at the study level using predefined criteria.

### 2.5. Statistical Analysis

For the included studies, data were extracted regarding author, study design, population, manual medicine technique(s) used, key findings, primary limitations, and risk of bias. Forest plots were generated using NetMetaEasy (Fekete et al., 2026), a web-based meta-analysis platform, to visualize pooled effect estimates [15]. Although NetMetaEasy supports network meta-analysis, only conventional pairwise random-effects meta-analyses were performed in the present study. Meta-analyses were performed using an inverse-variance random-effects model, with between-study variance estimated using the DerSimonian–Laird method. Continuous outcomes analyzed included time to first bowel movement, time to first flatus, and length of hospital stay where sufficient data were available. Where necessary, outcomes reported in hours were converted to days for consistency in quantitative synthesis. Pooled mean differences are reported with 95% confidence intervals, and statistical significance was assessed using the test for overall effect. Heterogeneity was assessed using the I^2^ statistic and the heterogeneity test *p*-value. For studies with multiple intervention arms, only intervention groups meeting the predefined eligibility criteria were included. Intervention arms were analyzed separately where appropriate, and control groups were not double-counted. For studies reporting medians and ranges or interquartile ranges, means and standard deviations were estimated using established conversion methods in accordance with Cochrane guidelines [16,17]. All statistical methods and analyses were independently reviewed for accuracy by a biostatistician.

### 2.6. Risk of Bias Assessment

Risk of bias was assessed using study design-specific appraisal tools: the Cochrane Risk of Bias Tool (RoB 2, version 2.0) was used for randomized controlled trials [18], the Risk of Bias in Non-randomized Studies of Interventions (ROBINS-I, version 1.0) tool was used for non-randomized interventional studies [19], and the Newcastle–Ottawa Scale (NOS, original version) was applied to observational and retrospective studies [20]. Risk of bias assessments were performed at the study level rather than separately for each reported outcome. Although RoB 2 and ROBINS-I can be applied on an outcome-specific basis, the present review utilized overall study-level judgments to provide a consistent assessment across studies with heterogeneous outcome reporting. Consequently, risk of bias may have differed between individual outcomes within a given study, and this should be considered when interpreting the findings. Certainty of evidence for outcomes included in quantitative synthesis was assessed using the Grading of Recommendations Assessment, Development, and Evaluation (GRADE) framework. Certainty ratings were determined by considering study limitations (risk of bias), inconsistency, indirectness, imprecision, and potential publication bias. Summary of Findings tables are provided in the Appendix A (Table A1) [21].

## 3. Results

### 3.1. Study Selection

A total of 537 unique records were identified in the initial literature search. After title and abstract screening, 22 articles were sought for retrieval and assessed for eligibility. These 22 articles underwent full-text review, and 12 of these studies were ultimately included in the review. Citation searching yielded five additional studies for inclusion. All included studies reported outcomes for postoperative bowel function in surgical patients following a therapeutic manual intervention. The study selection process is shown in Figure 1.

Ten articles were excluded: one study was excluded as a case report; three studies were excluded for evaluating acupuncture alone; two were excluded due to the use of electronic devices, which did not meet the definition of manual medicine; one study utilizing a rocking chair intervention was excluded; and three ongoing clinical trials were identified but excluded due to lack of available results [22,23,24]. While many articles addressed postoperative bowel dysfunction, relatively few evaluated manual techniques as a treatment modality. Although some studies assessed massage-based interventions, these were performed using mechanical devices rather than hands-on techniques and were therefore excluded based on the predefined eligibility criteria (e.g., Leblanc-Louvry et al.) [25].

### 3.2. Study Characteristics

The 17 included studies primarily investigated OMT (*n* = 9) and abdominal massage (*n* = 7) as non-pharmacologic interventions for postoperative bowel dysfunction. One additional study compared abdominal massage, acupressure, and control groups (*n* = 1).

Fourteen studies evaluated manual medicine as a prophylactic intervention to facilitate postoperative gastrointestinal recovery, whereas three studies evaluated manual medicine for the treatment of established postoperative bowel dysfunction or ileus. Across the included studies, the most frequently reported postoperative outcomes included time to first flatus (*n* = 7), time to first bowel movement or defecation (*n* = 8), and hospital length of stay (*n* = 8). Bowel sounds were reported in several studies but were not included in the quantitative synthesis due to heterogeneity in measurement, reporting, and concerns regarding their validity as an outcome measure. Additional relevant outcomes related to postoperative bowel dysfunction are synthesized narratively in the Discussion Section. The included studies, with their relevant characteristics and findings, are summarized in Table 1.

### 3.3. Risk of Bias

**Figure 2:**
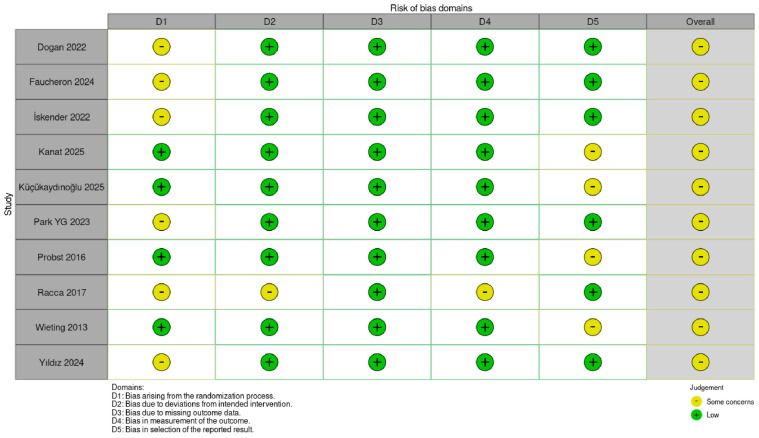
Risk of bias assessment of randomized controlled trials using the Cochrane Risk of Bias 2 (RoB 2) tool [32,33,34,35,36,37,38,39,41,42]. Risk of bias was evaluated across five domains: bias arising from the randomization process (D1), bias due to deviations from intended interventions (D2), bias due to missing outcome data (D3), bias in measurement of the outcome (D4), and bias in selection of the reported result (D5). Overall risk-of-bias judgments were assigned according to the RoB 2 algorithm. Abbreviations: RoB 2 = Cochrane Risk of Bias Tool for Randomized Trials.

**Figure 3:**
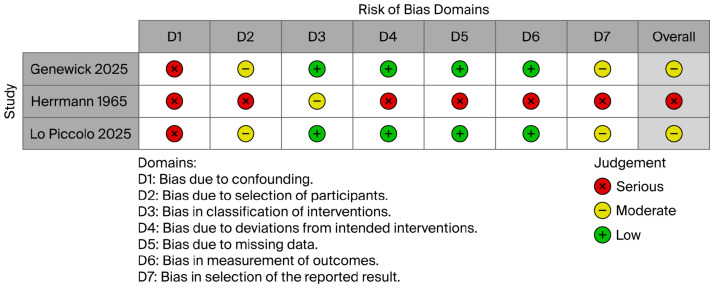
Risk of bias assessment of non-randomized interventional studies using the Risk of Bias in Non-randomized Studies of Interventions (ROBINS-I) tool [29,30,31]. Risk of bias was evaluated across seven domains: bias due to confounding (D1), bias in selection of participants into the study (D2), bias in classification of interventions (D3), bias due to deviations from intended interventions (D4), bias due to missing data (D5), bias in measurement of outcomes (D6), and bias in selection of the reported result (D7). Overall risk-of-bias judgments were assigned according to ROBINS-I guidance. Abbreviations: ROBINS-I = Risk of Bias in Non-randomized Studies of Interventions.

**Figure 4:**
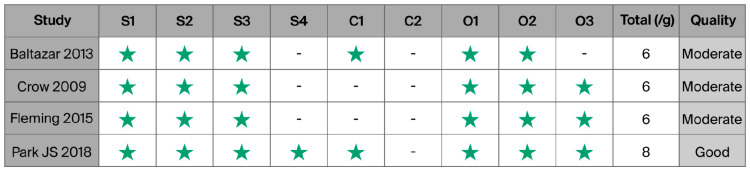
Methodological quality assessment of observational and retrospective cohort studies using the Newcastle–Ottawa Scale (NOS) [26,27,28,40]. Methodological quality was assessed using the Newcastle–Ottawa Scale across three domains: selection of study groups, comparability of groups, and ascertainment of outcomes. Studies were awarded stars for meeting predefined quality criteria, with higher scores indicating lower risk of bias and greater methodological rigor. Abbreviations: NOS = Newcastle–Ottawa Scale.

Risk of bias among randomized controlled trials (*n* = 10) was assessed using the Cochrane RoB2, with most studies demonstrating some concerns across domains, primarily in the randomization process and selection of reported results (Figure 2) [18]. Non-randomized interventional studies (*n* = 3), evaluated using ROBINS-I, showed an overall moderate to serious risk of bias, largely due to confounding and participant selection (Figure 3) [19]. Four observational studies were assessed using the Newcastle–Ottawa Scale. Three studies were rated as moderate methodological quality (6 stars), while one study was rated as good methodological quality (8 stars). Although these studies generally demonstrated favorable selection and outcome assessment domains, concerns regarding confounding, selection bias, and other limitations inherent to observational study designs remain (Figure 4) [20].

### 3.4. Study Outcomes

#### 3.4.1. Time to First Flatus

**Figure 5:**
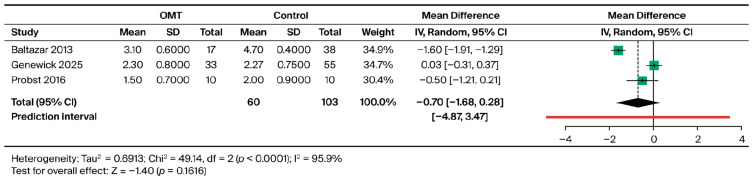
Effect of OMT on time to first flatus. Forest plot demonstrating the effect of OMT compared with control on time to first flatus using a random-effects model. Negative mean differences favor OMT. Included studies: [26,29,32]. Green squares represent individual study estimates with corresponding 95% confidence intervals; the black diamond represents the pooled effect estimate with its 95% confidence interval; the solid vertical black line indicates the line of no effect; the vertical dashed black line indicates the pooled effect estimate; and the red line indicates the prediction interval.

**Figure 6:**
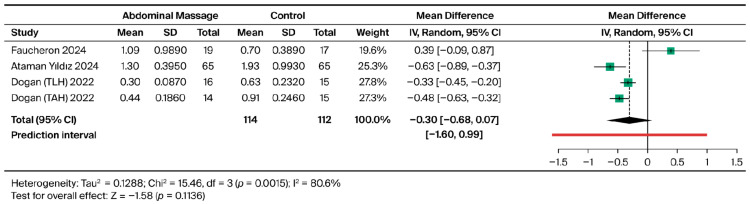
Effect of abdominal massage on time to first flatus. Forest plot demonstrating the effect of abdominal massage compared with control on time to first flatus using a random-effects model. Negative mean differences favor abdominal massage. Included studies: [35,36,42]. Green squares represent individual study estimates with corresponding 95% confidence intervals; the black diamond represents the pooled effect estimate with its 95% confidence interval; the solid vertical black line indicates the line of no effect; the vertical dashed black line indicates the pooled effect estimate; and the red line indicates the prediction interval.

Seven studies evaluated the impact of OMT (*n* = 3) and abdominal massage (*n* = 4) on time to first flatus (Figure 5 and Figure 6). Overall, findings were mixed across both intervention types.

Among the studies evaluating OMT, results were inconsistent: Genewick et al. reported no significant difference between OMT and control groups (2.30 vs. 2.27 days, *p* = 0.413) [29], whereas Baltazar et al. demonstrated a significantly shorter time to first flatus in patients receiving OMT compared with controls (3.1 vs. 4.7 days, *p* = 0.035) [26]. Probst et al. similarly observed a trend toward earlier flatus in the OMT group following abdominal surgery (1.5 vs. 2.0 days), although this difference did not reach statistical significance (*p* = 0.71) [32].

Findings for abdominal massage were similarly variable. Park et al. (2018) reported a shorter time to first flatus in the abdominal massage group compared with controls (34.3 vs. 41.3 h), although this finding was significant only on univariate analysis and not after multivariate adjustment [40]. Yildiz et al. demonstrated a significantly shorter time to first flatus in the intervention group (31.26 ± 9.48 vs. 46.41 ± 23.38 h, *p* < 0.001) [42], and Dogan et al. likewise reported a significant reduction in time to flatus following connective tissue massage in both total laparoscopic hysterectomy (TLH) and total abdominal hysterectomy (TAH) (*p* < 0.001) [35]. In contrast, a randomized controlled trial by Faucheron et al. found no significant benefit of abdominal massage following colorectal surgery (1065 vs. 1389 min, *p* = 0.274) and reported no differences in related postoperative outcomes [36].

#### 3.4.2. Time to First Bowel Movement

**Figure 7:**
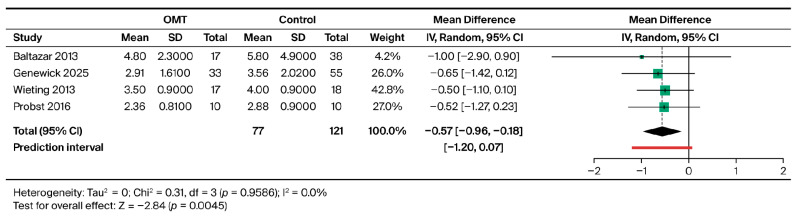
Effect of OMT on time to first bowel movement. Forest plot demonstrating the effect of OMT compared with control on time to first bowel movement using a random-effects model. Negative mean differences favor OMT. Included studies: [26,29,32,34]. Green squares represent individual study estimates with corresponding 95% confidence intervals; the black diamond represents the pooled effect estimate with its 95% confidence interval; the solid vertical black line indicates the line of no effect; the vertical dashed black line indicates the pooled effect estimate; and the red line indicates the prediction interval.

**Figure 8:**
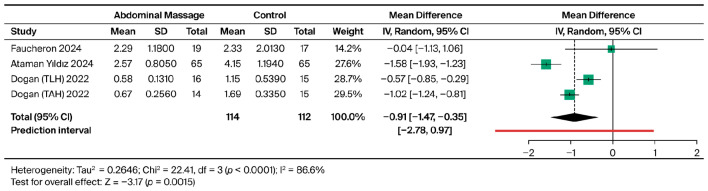
Effect of abdominal massage on time to first bowel movement. Forest plot demonstrating the effect of abdominal massage compared with control on time to first bowel movement using a random-effects model. Negative mean differences favor abdominal massage. Included studies: [35,36,42]. Green squares represent individual study estimates with corresponding 95% confidence intervals; the black diamond represents the pooled effect estimate with its 95% confidence interval; the solid vertical black line indicates the line of no effect; the vertical dashed black line indicates the pooled effect estimate; and the red line indicates the prediction interval.

Eight studies evaluated the impact of OMT and abdominal massage on the time to first bowel movement (Figure 7 and Figure 8). Overall, their results generally favored both interventions, although individual study findings were often not statistically significant.

Among studies evaluating OMT, all demonstrated a consistent trend toward earlier time to first bowel movement. Baltazar et al. reported a shorter time in the OMT group compared with controls (4.8 vs. 5.8 days, *p* = 0.43) [26], and Genewick et al. similarly found no significant difference between groups (2.91 vs. 3.56 days, *p* = 0.769) [29]. In postoperative coronary artery bypass graft patients, Wieting et al. observed a shorter time to first bowel movement in patients receiving OMT compared with placebo and standard care groups (3.5 vs. 4.0 vs. 4.0 days, *p* = 0.19) [34]. Probst et al. likewise reported a reduction following abdominal surgery (55.2 vs. 62.8 h, *p* = 0.43), although this did not reach statistical significance [32].

Findings for abdominal massage were also generally favorable. Yildiz et al. reported a significant decrease in time to first defecation in the intervention group compared with controls (61.6 vs. 99.5 h, *p* < 0.05) [42], and Dogan et al. similarly found significant reductions following connective tissue massage in both total TLH and TAH patient groups (*p* < 0.001) [35]. In contrast, Faucheron et al. found no significant difference between groups (*p* = 0.582) [36].

The substantial heterogeneity observed in the pooled analysis may reflect differences in the specific manual techniques employed, including connective tissue massage versus traditional abdominal massage, as well as variation in surgical populations, treatment protocols, and outcome measurement. Additionally, the relatively small number of studies available for quantitative synthesis limits the ability to perform informative subgroup or sensitivity analyses to further explore these sources of heterogeneity.

#### 3.4.3. Length of Hospital Stay

**Figure 9:**
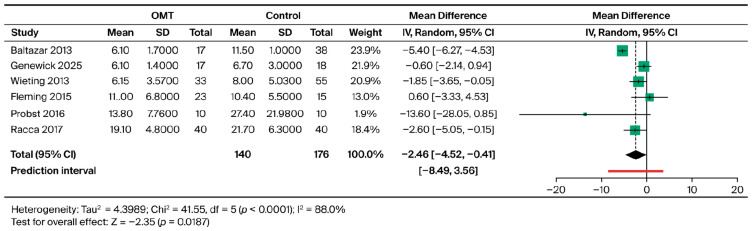
Effect of OMT on length of hospital stay. Forest plot demonstrating the effect of OMT compared with control on length of hospital stay using a random-effects model. Negative mean differences favor OMT. Included studies: [26,28,29,32,33,34]. Green squares represent individual study estimates with corresponding 95% confidence intervals; the black diamond represents the pooled effect estimate with its 95% confidence interval; the solid vertical black line indicates the line of no effect; the vertical dashed black line indicates the pooled effect estimate; and the red line indicates the prediction interval.

Eight studies in this review evaluated the impact of OMT (*n* = 8) on the length of hospital stay; however, only six provided sufficient quantitative data for inclusion in the meta-analysis (Figure 9). The studies by Crow et al. and Lo Piccolo et al. were included in the qualitative synthesis but were excluded from quantitative pooling because the necessary summary statistics were not reported in a format suitable for meta-analysis.

**Figure 10:**
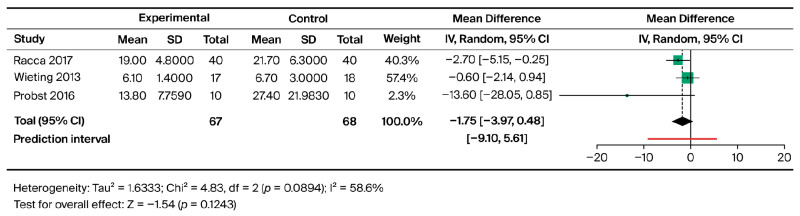
Sensitivity analysis of OMT on length of hospital stay restricted to randomized controlled trials. Forest plot demonstrating the effect of OMT compared with control on length of hospital stay using only randomized controlled trials and a random-effects model. Negative mean differences favor OMT. Included studies: [32,33,34]. Green squares represent individual study estimates with corresponding 95% confidence intervals; the black diamond represents the pooled effect estimate with its 95% confidence interval; the solid vertical black line indicates the line of no effect; the vertical dashed black line indicates the pooled effect estimate; and the red line indicates the prediction interval.

Because sufficient randomized data were available for this outcome, a sensitivity analysis restricted to randomized controlled trials was also performed to assess the robustness of the pooled estimate (Figure 10). Since an insufficient number of studies evaluating abdominal massage reported length of stay to permit quantitative synthesis, a forest plot was not generated for this outcome.

Several studies suggested that OMT may reduce the length of hospital stay. Baltazar et al. demonstrated a significantly shorter hospitalization in the OMT group compared with controls (6.1 vs. 11.5 days, *p* = 0.006), suggesting improved postoperative recovery [26]. Similarly, Crow et al. reported a significantly shorter length of stay among patients receiving OMT, with a mean reduction of 2.7 days (11.8 vs. 14.6 days, *p* = 0.029) [27]. A subgroup analysis by Genewick et al. also identified a statistically significant reduction in length of stay of approximately one day in patients undergoing open surgery (*p* = 0.011) [29]. Other studies demonstrated non-significant trends favoring OMT. Wieting et al. observed shorter hospital stays in CABG patients receiving OMT compared with placebo and standard care (6.1 vs. 6.3 vs. 6.7 days, *p* = 0.72) [34]. Probst et al. likewise reported a shorter mean length of stay in abdominal surgery patients treated with OMT (11.3 vs. 17.4 days, *p* = 0.29) [32]. Racca et al. similarly reported a significantly shorter hospitalization in patients receiving OMT compared with controls (19.1 vs. 21.7 days, *p* < 0.05) [33], while Lo Piccolo et al. found a reduction following appendectomy (4.6 vs. 7.0 days, *p* = 0.19) [31]. In contrast, Fleming et al. observed no benefit, reporting a slightly longer hospital stay in post-thoracotomy patients receiving OMT compared with controls (11.0 vs. 10.4 days, *p* = 0.90) [28].

## 4. Discussion

### 4.1. Osteopathic Manipulative Treatment

#### 4.1.1. Time to First Flatus

Overall, the pooled analysis of the time to first flatus did not demonstrate a statistically significant association with osteopathic manipulative treatment, but rather showed a trend toward earlier flatus that did not reach statistical significance (MD: −0.70 days, 95% CI [−1.68, 0.28]). Severe heterogeneity was observed across studies (I^2^ = 96%), limiting our interpretation. Individual study findings were mixed, ranging from a significant reduction in one study to no difference in another. The lack of statistical significance may reflect variability in study populations and surgical types, as well as differences in how flatus is reported, which may be less reliable and more patient-dependent compared with other gastrointestinal endpoints.

#### 4.1.2. Time to First Bowel Movement

In contrast, the pooled analysis of the time to first bowel movement demonstrated a statistically significant association with the use of OMT, showing a reduction in time to first bowel movement that reached statistical significance (MD: −0.57 days, 95% CI [−0.96, −0.18], *p* < 0.005), with no significant heterogeneity observed across studies. Notably, none of the individual studies reached statistical significance independently, despite all demonstrating a consistent trend toward earlier bowel movement in the OMT group. Notably, the prediction interval [−1.20, 0.07] marginally crosses zero despite the negligible heterogeneity (I^2^ = 0%), reflecting the wide t-multiplier associated with a prediction interval based on only four studies (2 degrees of freedom) rather than between-study inconsistency. Although the observed reduction in time to first bowel movement was relatively modest in magnitude (0.57 days), the return of bowel function is a clinically relevant postoperative milestone and may reflect earlier recovery of gastrointestinal motility. Whether this difference translates into meaningful patient-centered benefits, such as improved comfort, reduced postoperative complications, earlier discharge readiness, or lower healthcare utilization, remains uncertain, as these outcomes were not consistently reported among the included studies.

#### 4.1.3. Length of Hospital Stay

The pooled analysis of the effects of OMT on length of hospital stay showed a reduction in length of stay that reached statistical significance (MD: −2.46 days, 95% CI [−4.52, −0.41]), although substantial heterogeneity was observed across studies (I^2^ = 88%). Individual study results were variable, with some demonstrating significant reductions and others showing no difference or opposing trends. The prediction interval crosses zero, reflecting the substantial between-study heterogeneity; although the pooled effect is statistically significant, this heterogeneity must temper any conclusion, as the effect in a new setting could plausibly include no benefit. Despite this variability, most studies favored OMT, though one showed a small effect in the opposite direction.

A sensitivity analysis restricted to randomized controlled trials demonstrated a similar direction of effect favoring OMT (MD: −1.75 days, 95% CI [−3.97, 0.48]), though statistical significance was not maintained and the confidence interval crossed zero. Although the overall pooled analysis demonstrated a significant reduction in hospital length of stay associated with OMT, this finding was not maintained in the sensitivity analysis restricted to randomized controlled trials. This suggests that the observed benefit may be influenced, at least in part, by non-randomized studies and should therefore be interpreted cautiously. Heterogeneity was reduced compared to primary analysis (I^2^ = 58.6%), suggesting that differences in study design may contribute to variability in reported treatment effects. The observed reduction in length of stay may reflect broader aspects of postoperative recovery beyond gastrointestinal function alone. However, hospital length of stay is a multifactorial outcome influenced by numerous factors beyond gastrointestinal recovery, including surgical complexity, postoperative complications, baseline illness severity, institutional discharge criteria, Enhanced Recovery After Surgery (ERAS) protocols, and variations in hospital practice patterns. Consequently, while manual medicine may contribute to postoperative recovery, the observed reduction in length of stay likely reflects the influence of multiple clinical and institutional factors and should not be attributed solely to improvements in bowel function.

#### 4.1.4. Other Gastrointestinal Outcomes

Only one study directly reported postoperative ileus incidence: Herrmann described a markedly lower incidence of postoperative ileus among patients treated with OMT compared with controls (0.3% vs. 7.6%) [30]. However, these findings should be interpreted cautiously, as Herrmann was judged to have a serious risk of bias, indicating that this study should not be considered a primary contributor to the overall conclusions of this review. Other studies reported time to toleration of different types of diet. Baltazar et al. found a positive, but not significant, association with OMT shortening time to a clear liquid diet when compared to a control group (4.6 vs. 5.6 days, *p* = 0.59) [26]. In contrast, Probst et al. found no difference in median time to toleration of solid food between OMT and control groups (2 vs. 2 days, *p* = 0.32) [32]. Overall, the evidence for these additional gastrointestinal outcomes is limited and variable, precluding definitive conclusions.

### 4.2. Abdominal Massage

#### 4.2.1. Time to First Flatus

Evaluation of the pooled analysis of time to first flatus did not confirm a statistically significant association between abdominal massage and time to first flatus. The analysis showed a trend toward earlier flatus that did not reach statistical significance (MD: −0.30 days, 95% CI [−0.68, 0.07], *p* = 0.11). Substantial heterogeneity was seen across studies (I^2^ = 80.6%), limiting our interpretation. Faucheron et al.’s study was the only one to report a non-significant finding for abdominal massage and time to first flatus, and was notably the only study conducted exclusively with colorectal surgery patients. The asymmetry between the significant decrease in time to first bowel movement and non-significant decrease in time to first flatus may reflect differences in endpoint measurements, with time to first bowel movement being a more objectifiable clinical event than time to first flatus.

#### 4.2.2. Time to First Bowel Movement

In contrast, the pooled analysis of these four study arms showed a statistically significant decrease in time to first bowel movement (MD: −0.91, CI 95% [−1.47, −0.35], *p* = 0.0015). Notably, there was a large amount of heterogeneity between these studies (I^2^ = 86.6%), attributed mostly to differences in techniques and operations. While Yildiz et al. and Faucheron et al. used massage techniques focused in the abdomen, Dogan et al. used connective tissue massage on the posterior thorax and lumbosacral regions of the patient [35,36,42]. The type of surgery also varied between studies: Yildiz et al. analyzed cardiac surgery patients, Dogan et al. looked at hysterectomy patients, and Faucheron et al. included colorectal surgery patients [35,42]. These two variables, treatment location and surgery performed, add a significant heterogeneity that limits the generalizability of the pooled estimate. Faucheron et al.’s study was the only one with no statistical significance, at −0.04 [−1.13, 1.06], and was also the only study looking at surgery directly on the GI system. This non-significance could be explained by the bowel’s decreased responsiveness to abdominal massage and lower threshold to ileus [36]. The prediction interval extends past zero, largely reflecting the high heterogeneity, which limits any firm conclusion about the effect of abdominal massage on time to first bowel movement.

#### 4.2.3. Other Gastrointestinal Outcomes

Bowel sounds were reported in several studies evaluating abdominal massage. Kucukaydinoglu et al. found no significant difference in bowel sound scores between patients receiving abdominal massage and controls (*p* > 0.05) [39]. In contrast, Yildiz et al. reported a faster return of bowel sounds in the abdominal massage group, with 70.8% recovering by postoperative day one compared with 46.4% of controls, and fewer delays observed between postoperative days 3 and 5 [42]. Similarly, Iskender and Caliskan demonstrated greater improvement in bowel sounds over five postoperative days in the abdominal massage group compared with controls (−4.07 vs. −3.03, *p* = 0.02), and Kanat et al. reported a significantly shorter time to return of bowel sounds following abdominal massage (16.13 vs. 28.59 h, *p* < 0.001) [38]. Despite being commonly reported, bowel sounds were not included in quantitative synthesis due to the heterogeneity in measurement and reporting, as well as concerns regarding their validity as a surrogate for meaningful gastrointestinal recovery. Although historically used to assess the return of gastrointestinal function following surgery, bowel sounds have been shown to correlate poorly with clinically relevant endpoints such as time to first flatus and bowel movement [43,44,45].

Additional gastrointestinal outcomes were variably reported. Park et al. (2023) found that fewer constipation remedies were required in patients receiving abdominal massage compared with controls (9 vs. 15, *p* = 0.049), although no significant differences were observed in postoperative ileus or length of hospital stay [41]. Iskender and Caliskan also reported earlier first postoperative day defecation in the abdominal massage group compared with controls, though the effect was less pronounced than in the acupressure group [37]. However, the study by Iskender and Caliskan was not included in pooled analyses because time to first defecation was reported as the distribution of patients across postoperative days rather than as summary statistics suitable for meta-analysis. Overall, these additional outcomes demonstrated substantial heterogeneity in both measurement and clinical relevance, limiting their utility for quantitative synthesis and interpretation of consistent associations.

### 4.3. Summary

Across included studies, manual medicine interventions were associated with improvements in postoperative bowel recovery, most consistently with time to first bowel movement. However, these findings should be interpreted cautiously, as several included studies, particularly non-randomized investigations, were at a moderate to serious risk of bias, and residual confounding and selection bias may have influenced the observed treatment effects. Consequently, the reported associations should not be interpreted as definitive evidence of a causal relationship. Both OMT and abdominal massage showed associations with earlier return of bowel function. Notably, the OMT pooled analysis showed statistical significance for time to first bowel movement, with no detectable heterogeneity (I^2^ = 0%). Although OMT was found to statistically significantly decrease length of stay by 2.46 days, the high heterogeneity and prediction interval that includes no benefit warrant cautious interpretation. Time to first flatus did not reach significance for either modality, an outcome likely influenced by its substantial heterogeneity and the variability inherent in measuring flatus. Taken together, these findings suggest that manual medicine may have a role as an adjunctive approach in postoperative care, particularly for facilitating bowel recovery, although the available evidence is limited by heterogeneity and the small number of studies available for several outcomes. The identification of ongoing clinical trials evaluating abdominal massage in postoperative patients further suggests sustained research interest and may help clarify the role of these interventions across additional clinical outcomes [22,23,24].

### 4.4. Limitations

Several limitations were identified within the included studies, affecting the strength of the findings. Several studies had small sample sizes: for example, Baltazar et al. had a total population of 55 patients, and only 17 patients received OMT during the study [26]. There was a significant heterogeneity in study design, patient populations, and interventions between the included studies. Protocols for OMT and abdominal massage varied considerably between studies and were often poorly described. Furthermore, reported outcome measures were heterogeneous, with many studies using different endpoints ranging from time to first flatus to length of hospital stay. Additionally, outcome definitions and measurement methods were not standardized across studies, which may affect comparability. Risk of bias was an important consideration in the included studies. For instance, Herrmann was found to have a serious risk of bias, as determined using ROBINS-I [20,30]. This study was not included in our quantitative synthesis, as it did not report outcomes aligned with those selected for meta-analysis; instead, its findings were synthesized narratively. While its exclusion from the pooled analysis limited its influence on our quantitative estimates, the consideration of study-level bias remains important when interpreting overall evidence. Additionally, variations in massage technique and location between studies add heterogeneity. For example, Dogan et al. used a massage technique focused on the back and flank of the patient, whereas other studies treated only the abdomen [35]. Manual therapy interventions are inherently difficult to blind, which increases the risk of performance bias. Small sample sizes and limited efforts at allocation concealment in several of the included studies further constrain interpretation of the reported effects. These factors reduce confidence in the study findings and limit the strength of conclusions regarding clinical significance.

This systematic review also has limitations. Although multiple major databases, trial registries, and citation searches were utilized to maximize study identification, the literature search did not include Embase, Scopus, Web of Science, or CINAHL. The omission of these sources may have resulted in missed relevant studies and could have affected the comprehensiveness of the evidence base. Inter-reviewer agreement statistics were not prospectively recorded during study screening, so formal measures of screening reliability such as Cohen’s kappa could not be calculated. The inclusion of multiple types of manual medicine may limit generalizability, and variability in massage form, OMT techniques, and intervention duration contributed to both clinical and methodological heterogeneity. An additional limitation is the clinical heterogeneity of the included populations. The analyzed studies encompassed a variety of surgical procedures, including cardiac, abdominal, colorectal, gynecologic, and thoracic operations. Although all studies evaluated postoperative bowel dysfunction or recovery-related outcomes following surgery, differences in surgical approach, baseline patient characteristics, and expected gastrointestinal recovery time between studies may have influenced treatment effects. Consequently, the pooled estimates should be interpreted with caution, as the magnitude of the benefit associated with manual therapies may vary across surgical populations. While the inclusion of diverse surgical cohorts improves the potential generalizability across surgical operative settings, it reduces the precision of effect estimates within individual surgical specialties. As the quantitative synthesis included non-randomized studies, the pooled estimates reflect associations and should not be interpreted as evidence of a causal effect. The small number of studies included in each quantitative analysis limits statistical power and increases the influence of individual studies on pooled estimates. A limitation of this analysis is the exclusion of Crow et al., the only study reporting adjusted outcome measures, from pooled analysis, although statistical significance was maintained despite its exclusion [27]. Furthermore, the need to estimate mean and standard deviation for Probst et al. may introduce additional variability and contribute to heterogeneity, as reflected by the large calculated standard deviation in the control group (21.98 days) [32]. Additionally, although several included studies reported no intervention-related adverse events, adverse event reporting was not consistently or systematically assessed across all studies. Consequently, the present review was not designed to formally evaluate the safety profile of manual medicine interventions.

This review was conducted in accordance with PRISMA reporting guidelines but was not prospectively registered, which may limit its methodological transparency. Despite the identification of numerous relevant records, some potentially informative studies, such as that of Leblanc-Louvry, were excluded because abdominal massage was delivered mechanically rather than manually [25]. Similarly, studies utilizing acupuncture as the sole intervention were excluded, as they fell outside of the predefined eligibility criteria for manual therapy in this review. Additional limitations include the inability to formally assess publication bias, as most pooled analyses included fewer than ten studies, a threshold below which funnel plots and statistical tests for asymmetry are generally considered unreliable and potentially misleading. Risk of bias assessments were conducted at the study level rather than for individual outcomes. Because outcome measurement, missing data, and reporting practices may vary across outcomes within the same study, the risk of bias associated with specific endpoints, such as time to first flatus, time to first bowel movement, and length of hospital stay, may not be fully captured by the overall study-level assessments. Additionally, because no single primary outcome was prespecified, the review evaluated multiple indicators of postoperative gastrointestinal recovery. As a result, the findings should be interpreted as exploratory, particularly for outcomes supported by a limited number of studies.

Consequently, the overall certainty of evidence supporting these findings remains limited, and the reported associations should be interpreted cautiously.

## 5. Conclusions

While the available evidence suggests that manual medicine interventions may aid postoperative bowel recovery, the strength of this association varied across outcomes. The strongest association was observed between the use of OMT and a reduction in time to first bowel movement, which reaches statistical significance with no detectable heterogeneity (I^2^ = 0%). Abdominal massage was also significantly associated with a reduced time to first bowel movement, although this conclusion was limited by high heterogeneity and a prediction interval that includes no benefit. OMT was associated with a statistically significant reduction in hospital length of stay, though this correlation was also limited by heterogeneity. Time to first flatus did not reach statistical significance with either modality and was characterized by severe heterogeneity across the included studies, possibly reflecting variability across surgical populations and less reliable endpoints, such as patient-reported time to first flatus.

The patterns of statistically significant associations differed across outcomes, with OMT demonstrating associations with both reduced time to first bowel movement and reduced hospital length of stay, whereas abdominal massage reached a significant association with time to first bowel movement alone. Although these findings may reflect genuine variations between osteopathic manipulative treatment and abdominal massage, they may also arise from the limited number of trials, differences in the outcomes measured, and heterogeneity throughout.

These findings are tempered by important limitations: few studies per outcome, substantial heterogeneity in surgical populations and treatment techniques, and variability in outcome definitions. Further trials with larger patient populations are warranted to better define the magnitude and consistency of these associations.

## Figures and Tables

**Table 1:** Summary of included studies evaluating non-pharmacologic manual medicine techniques for the management of postoperative bowel dysfunction.

Author (Year)	Study Design	Population (*n*)	Surgical Procedure	Non-Pharmacologic Technique(s) Used	Comparator	Clinical Context	Key Findings
Baltazar et al. [26]	Retrospective cohort	55 patients	Small or large bowel resection, gastric resection, or repair	OMT	Standard postoperative care	Prevention	OMT was associated with significantly earlier return of flatus and a markedly shorter postoperative hospital length of stay, suggesting improved postoperative gastrointestinal recovery.
Crow et al. [27]	Retrospective cohort	331 patients	Abdominal surgery	OMT	No OMT	Treatment	Patients receiving OMT had a significantly shorter hospital length of stay compared with controls.
Fleming et al. [28]	Retrospective cohort study	38 patients	Posterolateral thoracotomy	OMT	Standard postoperative care	Prevention	No significant reduction in hospital length of stay was observed; interpretation was limited by greater baseline illness severity in the OMT group, including higher rates of ICU admission and postoperative ileus.
Genewick et al. [29]	Non-randomized QI study	88 patients	Colorectal and abdominal surgery	OMT	Standard postoperative care	Prevention	No significant differences were observed in time to flatus or first bowel movement; however, OMT was associated with a modest reduction in length of stay, with subgroup analysis demonstrating significant benefit in patients undergoing open surgery.
Herrmann [30]	Retrospective observational study	317 patients	Major surgery	OMT	Usual postoperative care	Treatment	There was an association between OMT and improved postoperative gastrointestinal outcomes, including lower rates of postoperative ileus and earlier return of bowel function; however, these findings should be interpreted cautiously due to the study’s serious risk of bias.
Lo Piccolo et al. [31]	Non-randomized time-controlled clinical trial	43 patients	Laparoscopic appendectomy	OMT	Standard postoperative care	Prevention	OMT was associated with shorter hospital length of stay, improved pain control, and favorable trends toward earlier bowel recovery and mobilization, supporting its potential role in enhancing postoperative recovery.
Probst et al. [32]	Randomized controlled trial	20 patients	Elective bowel resection	OMT	Sham OMT	Prevention	OMT was feasible, with no intervention-related adverse events reported. The study also demonstrated reduced postoperative pain and favorable trends toward earlier return of bowel function and shorter hospital stay.
Racca et al. [33]	Randomized controlled trial	80 patients	CABG, valve surgery, or ascending aortic surgery	OMT	Standard rehabilitation	Prevention	OMT was associated with a significantly shorter postoperative length of stay compared to standard rehabilitation alone, suggesting a potential benefit in overall postoperative recovery.
Wieting et al. [34]	Randomized controlled trial	53 patients	Coronary artery bypass graft	OMT	Placebo OMT and standard care	Prevention	OMT was associated with shorter hospital stay, earlier return of bowel function, and improved functional recovery, though differences did not reach statistical significance in this pilot study.
Dogan et al. [35]	Randomized controlled trial	60 patients	Total laparoscopic hysterectomy or total abdominal hysterectomy	Abdominal massage	Standard postoperative care	Prevention	Abdominal massage was associated with reduced postoperative pain, decreased analgesic requirements, and earlier return of flatus and defecation, with greater functional improvement observed in laparoscopic patients.
Faucheron et al. [36]	Randomized controlled trial	36 patients	Resection of small bowel, colon, or high rectum with anastomosis	Abdominal massage	Standard postoperative care	Prevention	No significant differences were observed in time to first flatus, bowel movement, pain, or quality-of-life measures between abdominal massage and control groups.
İskender & Çalışkan [37]	Randomized controlled trial	91 patients	Total knee arthroplasty	Abdominal massage	Standard care (and acupressure arm)	Prevention	Both abdominal massage and acupressure were associated with reduced constipation severity and earlier defecation compared with standard care.
Kanat & Uğraş [38]	Randomized controlled trial	78 patients	Partial hip replacement	Abdominal massage	Standard postoperative care	Prevention	Patients receiving abdominal massage demonstrated earlier recovery of bowel activity and improved postoperative pulmonary function compared with controls.
Küçükaydınoğlu & Turan [39]	Randomized controlled trial	68 patients	Not reported	Abdominal massage	Pharmacologic therapy	Treatment	Abdominal massage produced gastrointestinal symptom outcomes comparable to pharmacologic therapy and was associated with improved patient comfort.
Park et al. 2018 [40]	Observational study	716 patients	Robot-assisted laparoscopic prostatectomy	Abdominal massage	No abdominal massage	Prevention	Abdominal massage was associated with a shorter time to first flatus following robot-assisted laparoscopic prostatectomy on univariate analysis, though this association was not maintained after multivariate adjustment.
Park et al., 2023 [41]	Randomized controlled trial	88 patients	Internal fixation or arthroplasty of hip	Abdominal massage	Standard postoperative care	Prevention	Abdominal massage was associated with reduced constipation-related interventions and lower rates of defecation failure, though no significant effect was observed on postoperative ileus or length of stay.
Yıldız et al. [42]	Randomized controlled trial	130 patients	Cardiac surgery	Abdominal massage	Standard postoperative care	Prevention	Abdominal massage was associated with earlier return of bowel sounds, faster passage of flatus, and earlier defecation compared with standard postoperative care.

This table summarizes the characteristics and findings of studies included in this systematic review. Studies were identified through a comprehensive literature search of MEDLINE/PubMed, Google Scholar, the Cochrane Library, and Semantic Scholar. Included studies evaluated the use of manual therapy as a non-pharmacologic adjunct for the management of postoperative bowel dysfunction. For each study, the study design, population size, technique employed, and key findings are reported. Study designs ranged from retrospective cohorts and chart reviews to randomized controlled trials and observational studies. OMT: osteopathic manipulative treatment; QI: quality improvement.

## Data Availability

The data presented in this study are derived from publicly available sources. The datasets analyzed were obtained from MEDLINE/PubMed, Google Scholar, Semantic Scholar, the Cochrane Library, and ClinicalTrials.gov.

## Supplementary Materials

The following supporting information can be downloaded at: https://www.mdpi.com/article/10.3390/jcm15135245/s1. Supplementary File S1: PRISMA 2020 Checklist; Supplementary File S2: PRISMA 2020 for Abstracts Checklist.

## Abbreviations

The following abbreviations are used in this manuscript:

Abbreviation	Full Term
CABG	Coronary Artery Bypass Graft
CI	Confidence Interval
ERAS	Enhanced Recovery After Surgery
GI	Gastrointestinal
GRADE	Grading of Recommendations Assessment, Development, and Evaluation
ICU	Intensive Care Unit
I^2^	I-Squared (Measure of Heterogeneity)
MD	Mean Difference
MeSH	Medical Subject Headings
NMA	Network Meta-Analysis
NOS	Newcastle–Ottawa Scale
OMT	Osteopathic Manipulative Treatment
PRISMA	Preferred Reporting Items for Systematic Reviews and Meta-Analyses
QI	Quality Improvement
RCT	Randomized Controlled Trial
RoB 2	Risk of Bias Tool 2
ROBINS-I	Risk of Bias in Non-randomized Studies of Interventions
TAH	Total Abdominal Hysterectomy
TLH	Total Laparoscopic Hysterectomy

## Appendix A

The MEDLINE/PubMed search combined text words and Medical Subject Headings (MeSH) as follows:

(“Postoperative Ileus”[Mesh] OR “postoperative ileus”[Title/Abstract] OR “paralytic ileus”[Title/Abstract] OR “postoperative bowel dysfunction”[Title/Abstract] OR “postoperative gastrointestinal dysfunction”[Title/Abstract] OR “delayed gastrointestinal recovery”[Title/Abstract] OR “postoperative constipation”[Title/Abstract] OR “bowel motility”[Title/Abstract] OR “gastrointestinal transit”[Title/Abstract] OR “gut motility”[Title/Abstract]) AND (“Massage”[Mesh] OR “Manipulation, Osteopathic”[Mesh] OR “Manipulation, Spinal”[Mesh] OR “Musculoskeletal Manipulations”[Mesh] OR massage[Title/Abstract] OR “abdominal massage”[Title/Abstract] OR “massage therapy”[Title/Abstract] OR osteopathic[Title/Abstract] OR “osteopathic manipulative treatment”[Title/Abstract] OR “osteopathic manipulation”[Title/Abstract] OR chiropractic[Title/Abstract] OR “spinal manipulation”[Title/Abstract] OR “manual therapy”[Title/Abstract] OR “visceral manipulation”[Title/Abstract] OR “myofascial release”[Title/Abstract] OR “soft tissue mobilization”[Title/Abstract]) AND (postoperative[Title/Abstract] OR postsurgical[Title/Abstract] OR surgery[Title/Abstract] OR surgical[Title/Abstract]).

The Google Scholar search used the query (“postoperative ileus” OR “paralytic ileus” OR “postoperative bowel dysfunction” OR “postoperative gastrointestinal dysfunction” OR “postoperative constipation” OR “delayed gastrointestinal recovery”) AND (“abdominal massage” OR “massage therapy” OR massage OR “osteopathic manipulation” OR “osteopathic manipulative treatment” OR “osteopathic manipulative medicine” OR chiropractic OR “spinal manipulation” OR “manual therapy” OR “visceral manipulation” OR “myofascial release” OR “soft tissue mobilization”).

The Semantic Scholar search used (“postoperative ileus” OR “postoperative bowel dysfunction” OR “postoperative constipation”)

AND

(“manual therapy” OR massage OR osteopathic OR chiropractic OR “visceral manipulation”)”.

The Cochrane Library search used (“postoperative ileus” OR “paralytic ileus” OR “postoperative bowel dysfunction” OR “postoperative gastrointestinal dysfunction” OR “postoperative constipation” OR “delayed gastrointestinal recovery”)

AND

(massage OR “abdominal massage” OR “massage therapy” OR osteopathic OR “osteopathic manipulative treatment” OR chiropractic OR “spinal manipulation” OR “manual therapy” OR “visceral manipulation” OR “myofascial release”).

The search of ClinicalTrials.gov used “postoperative ileus OR postoperative bowel dysfunction OR paralytic ileus OR postoperative constipation” as the condition/disease and “massage OR “abdominal massage” OR “massage therapy” OR osteopathic OR “osteopathic manipulative treatment” OR chiropractic OR “spinal manipulation” OR “manual therapy” OR “visceral manipulation” OR “myofascial release”” in the other terms selection.

**Table A1:** Summary of findings and GRADE certainty assessment.

Outcome	Studies (*n*)	Participants (*n*)	Effect Estimate (95% CI)	Certainty of Evidence (GRADE)	Reasons for Downgrading
OMT—Time to first bowel movement	4	198	MD −0.57 days (−0.96 to −0.18)	Moderate	Downgraded one level for risk of bias due to some concerns across included randomized and non-randomized studies.
Abdominal massage—Time to first bowel movement	4	226	MD −0.91 days (−1.47 to −0.35)	Low	Downgraded one level for risk of bias and one level for substantial inconsistency (I^2^ = 86.6%).
OMT—Time to first flatus	3	163	MD −0.70 days (−1.68 to 0.28)	Very Low	Downgraded for risk of bias, very serious inconsistency (I^2^ = 95.9%), and imprecision due to wide confidence intervals crossing the null effect.
Abdominal massage—Time to first flatus	4	226	MD −0.30 days (−0.68 to 0.07)	Very Low	Downgraded for risk of bias, substantial inconsistency (I^2^ = 80.6%), and imprecision due to confidence intervals crossing the null effect.
OMT—Length of hospital stay	6	316	MD −2.46 days (−4.52 to −0.41)	Low	Downgraded for risk of bias and substantial inconsistency (I^2^ = 88.0%). Sensitivity analysis restricted to randomized controlled trials did not maintain statistical significance.

Abbreviations: GRADE = Grading of Recommendations Assessment, Development, and Evaluation; OMT = osteopathic manipulative treatment; MD = mean difference; CI = confidence interval.

## References

[1] Bragg D., El-Sharkawy A.M., Psaltis E., Maxwell-Armstrong C.A., Lobo D.N. (2015). Postoperative ileus: Recent developments in pathophysiology and management. Clin. Nutr..

[2] Venara A., Neunlist M., Slim K., Barbieux J., Colas P., Hamy A., Meurette G. (2016). Postoperative ileus: Pathophysiology, incidence, and prevention. J. Visc. Surg..

[3] Harnsberger C.R., Maykel J.A., Alavi K. (2019). Postoperative Ileus. Clin. Colon Rectal Surg..

[4] Wells C.I., Milne T.G.E., Seo S.H.B., Chapman S.J., Vather R., Bissett I.P., O’GRady G. (2022). Post-operative ileus: Definitions, mechanisms and controversies. ANZ J. Surg..

[5] Ripollés-Melchor J., Ramírez-Rodríguez J.M., Casans-Francés R., Aldecoa C., Abad-Motos A., Logroño-Egea M., García-Erce J.A., Camps-Cervantes Á., Ferrando-Ortolá C., de la Rica A.S. (2019). Association Between Use of Enhanced Recovery After Surgery Protocol and Postoperative Complications in Colorectal Surgery: The Postoperative Outcomes Within Enhanced Recovery After Surgery Protocol (POWER) Study. JAMA Surg..

[6] Vaughan-Shaw P.G., Fecher I.C., Harris S., Knight J.S. (2012). A meta-analysis of the effectiveness of the opioid receptor antagonist alvimopan in reducing hospital length of stay and time to GI recovery in patients enrolled in a standardized accelerated recovery program after abdominal surgery. Dis. Colon Rectum.

[7] Ljungqvist O., Scott M., Fearon K.C. (2017). Enhanced Recovery After Surgery: A Review. JAMA Surg..

[8] Short V., Herbert G., Perry R., Atkinson C., Ness A.R., Penfold C., Thomas S., Andersen H.K., Lewis S.J. (2015). Chewing gum for postoperative recovery of gastrointestinal function. Cochrane Database Syst. Rev..

[9] De Castro S.M.M., Van den Esschert J.W., Van Heek N.T., Dalhuisen S., Koelemay M.J.W., Busch O.R.C., Gouma D.J. (2008). A systematic review of the efficacy of gum chewing for the amelioration of postoperative ileus. Dig. Surg..

[10] Henwood L., Le Donne M.E., Vaughn A., Kamil S., Harrington A., Scott R. (2024). The Effects of Osteopathic Manipulative Treatment (OMT) on Postoperative Length of Stay: A Meta-Analysis. Cureus.

[11] Randall C.G., Paul H.A., Lumley H., Ortega A., Rowley J., Brown B., Mohan S., Smith K., Messer T., Swan E. (2024). Osteopathic Manipulative Treatment During Post-operative Recovery: A Scoping Review. Cureus.

[12] Ghotra K., Iyer A., Uberti J., Armstrong N., Lai S., Whittaker A., Scherer T., Iyer K. (2026). Role of osteopathic manipulative treatment in perioperative pain management: A systematic review with exploratory meta-analysis. Cureus.

[13] Sinclair M. (2011). The use of abdominal massage to treat chronic constipation. J. Bodyw. Mov. Ther..

[14] Page M.J., McKenzie J.E., Bossuyt P.M., Boutron I., Hoffmann T.C., Mulrow C.D., Shamseer L., Tetzlaff J.M., Akl E.A., Brennan S.E. (2021). The PRISMA 2020 statement: An updated guideline for reporting systematic reviews. BMJ.

[15] Fekete J.T., Komócsi A., Győrffy B. (2026). NetMetaEasy: Enabling rapid and user-friendly network meta-analysis (NMA) for comparative effectiveness research. Br. J. Pharmacol..

[16] Cochrane Collaboration (2023). Cochrane Handbook for Systematic Reviews of Interventions.

[17] Wan X., Wang W., Liu J., Tong T. (2014). Estimating the sample mean and standard deviation from the sample size, median, range and/or interquartile range. BMC Med. Res. Methodol..

[18] Sterne J.A.C., Savović J., Page M.J., Elbers R.G., Blencowe N.S., Boutron I., Cates C.J., Cheng H.Y., Corbett M.S., Eldridge S.M. (2019). RoB 2: A revised tool for assessing risk of bias in randomized trials. BMJ.

[19] Sterne J.A., Hernán M.A., Reeves B.C., Savović J., Berkman N.D., Viswanathan M., Henry D., Altman D.G., Ansari M.T., Boutron I. (2016). ROBINS-I: A tool for assessing risk of bias in non-randomised studies of interventions. BMJ.

[20] Wells G.A., Shea B., O’Connell D., Peterson J., Welch V., Losos M., Tugwell P. (2000). The Newcastle–Ottawa Scale (NOS) for Assessing the Quality of Nonrandomized Studies in Meta-Analyses.

[21] Guyatt G.H., Oxman A.D., Vist G.E., Kunz R., Falck-Ytter Y., Alonso-Coello P., Schünemann H.J. (2008). GRADE: An emerging consensus on rating quality of evidence and strength of recommendations. BMJ.

[22] Ma Y., Wang H., Liu S., Xu Z., Cao Y., Wang G., Liu X., Zhang K., Guo G. (2026). Effectiveness of self-abdominal massage on gastrointestinal function in postoperative ileus: A randomized controlled trial protocol. BMJ Open.

[23] Ozkeskin M. The Effect of Preoperative Abdominal Superficial Effleurage Training on the Development of Postoperative Constipation in Patients Undergoing Abdominal Surgery. ClinicalTrials.gov Identifier: NCT07344532. Updated 27 January 2026. NCT07344532.

[24] Sarı M. The Effect of Abdominal Massage and Warm Water Consumption on Postoperative Constipation Development and Quality of Recovery in Patients Undergoing Hip Fractures Surgery: A Randomized Comparative Study. ClinicalTrials.gov Identifier: NCT07437014. Updated 4 March 2026. NCT07437014.

[25] Le Blanc-Louvry I., Costaglioli B., Boulon C., Leroi A.M., Ducrotte P. (2002). Does mechanical massage of the abdominal wall after colectomy reduce postoperative pain and shorten the duration of ileus? Results of a randomized study. J. Gastrointest. Surg..

[26] Baltazar G.A., Betler M.P., Akella K., Khatri R., Asaro R., Chendrasekhar A. (2013). Effect of osteopathic manipulative treatment on incidence of postoperative ileus and hospital length of stay in general surgical patients. J. Am. Osteopath. Assoc..

[27] Crow W.T., Gorodinsky L. (2009). Does osteopathic manipulative treatment (OMT) improve outcomes in patients who develop postoperative ileus? A retrospective chart review. Int. J. Osteopath. Med..

[28] Fleming R.K., Snider K.T., Blanke K.J., Johnson J.C. (2015). The effect of osteopathic manipulative treatment on length of stay in posterolateral postthoracotomy patients: A retrospective case note study. Int. J. Osteopath. Med..

[29] Genewick J., Amendolara A., Robinson S., McDonough M., Loera M., Stacey S.K. (2025). Osteopathic Manipulative Treatment Protocol for Postoperative Care Following Abdominal Surgery: A Quality Improvement Project. Cureus.

[30] Herrmann E.P. (1965). Postoperative adynamic ileus: Its prevention and treatment by osteopathic manipulation. DO.

[31] Lo Piccolo R., Cantagalli M.M., Ferroni T., Fracchiolla F., Morabito A. (2025). The effect of osteopathic manipulative treatment on length of stay and pain relief in pediatric appendectomy: A pilot non-randomized time-controlled clinical trial. Front. Pediatr..

[32] Probst P., Büchler E., Doerr-Harim C., Knebel P., Thiel B., Ulrich A., Diener M.K. (2016). Randomised controlled pilot trial on feasibility, safety and effectiveness of osteopathic manipulative treatment following major abdominal surgery (OMANT pilot trial). Int. J. Osteopath. Med..

[33] Racca V., Bordoni B., Castiglioni P., Modica M., Ferratini M. (2017). Osteopathic manipulative treatment improves heart surgery outcomes: A randomized controlled trial. Ann. Thorac. Surg..

[34] Wieting J.M., Beal C., Roth G.L., Gorbis S., Dillard L., Gilliland D., Rowan J. (2013). The effect of osteopathic manipulative treatment on postoperative medical and functional recovery of coronary artery bypass graft patients. J. Am. Osteopath. Assoc..

[35] Dogan H., Demir Caltekin M., Gunal A. (2022). Short-term effects of connective tissue massage after hysterectomy: A randomized controlled study. J. Manip. Physiol. Ther..

[36] Faucheron J.-L., Vincent D., Barbut M., Jacquet-Perrin I., Sage P.-Y., Foote A., Bellier A., Quesada J.-L., Tidadini F., Trilling B. (2024). Abdominal massage to prevent ileus after colorectal surgery: A single-center, prospective, randomized clinical trial: The MATRAC Trial. Tech. Coloproctol..

[37] İskender M., Çalışkan N. (2022). Effect of acupressure and abdominal massage on constipation in patients with total knee arthroplasty: A randomized controlled study. Clin. Nurs. Res..

[38] Kanat C., Altun Uğraş G. (2026). Effects of abdominal massage on bowel activity and pulmonary function test results in older patients undergoing partial hip replacement: A randomized controlled trial. J. Perianesth. Nurs..

[39] Küçükaydınoğlu S., Turan N. (2025). The effect of abdominal massage applied after surgery on gastrointestinal functions and comfort level: A randomized controlled study. Perioper. Med..

[40] Park J.S., Kim J., Jang W.S., Heo J.E., Elghiaty A., Rha K.H., Choi Y.D., Ham W.S. (2018). Management of postoperative ileus after robot-assisted laparoscopic prostatectomy. Medicine.

[41] Park Y.G., Kim B.S., Kang K.T., Ha Y.C. (2023). Effects of abdominal massage for preventing acute postoperative constipation in hip fractures: A prospective interventional study. Clin. Orthop. Surg..

[42] Yıldız G., Çınar F., Kadiroğulları E., Eti Aslan F. (2024). Effect of abdominal massage on constipation after cardiac surgery. J. Educ. Res. Nurs..

[43] Chapman S.J., Thorpe G., Vallance A.E., Harji D.P., Lee M.J., Fearnhead N.S. (2018). Systematic review of definitions and outcome measures for return of bowel function after gastrointestinal surgery. BJS Open.

[44] Read T.E., Brozovich M., Andujar J.E., Ricciardi R., Caushaj P.F. (2017). Bowel Sounds Are Not Associated with Flatus, Bowel Movement, or Tolerance of Oral Intake in Patients After Major Abdominal Surgery. Dis. Colon Rectum.

[45] Drake A., Franklin N., Schrock J.W., Jones R.A. (2021). Auscultation of Bowel Sounds and Ultrasound of Peristalsis Are Neither Compartmentalized nor Correlated. Cureus.

