# Supplementary material for: Non-Pharmacologic Manual Therapies for Postoperative Bowel Dysfunction: A Systematic Review and Meta-Analysis
Source: J Clin Med. 2026 Jul 4;15(13):5245. doi: 10.3390/jcm15135245 (PMC13363015; doi:10.3390/jcm15135245)
Supplement: Supplementary file 1 [file jcm-15-05245-s001.zip › Supplementary File S1 PRISMA 2020 Checklist.pdf]

PRISMA 2020 Checklist

Section and Topic	Item #	Checklist item	Location where item is reported
**TITLE**			
Title	1	Identify the report as a systematic review.	Title
**ABSTRACT**			
Abstract	2	See the PRISMA 2020 for Abstracts checklist.	Abstract
**INTRODUCTION**			
Rationale	3	Describe the rationale for the review in the context of existing knowledge.	Introduction paragraph on postoperative bowel dysfunction
Objectives	4	Provide an explicit statement of the objective(s) or question(s) the review addresses.	Final sentence of intro
**METHODS**			
Eligibility criteria	5	Specify the inclusion and exclusion criteria for the review and how studies were grouped for the syntheses.	Methods → Eligibility Criteria section
Information sources	6	Specify all databases, registers, websites, organisations, reference lists and other sources searched or consulted to identify studies. Specify the date when each source was last searched or consulted.	Materials and Methods → Information Sources and Search Strategy (March 17, 2026)
Search strategy	7	Present the full search strategies for all databases, registers and websites, including any filters and limits used.	Materials and Methods → Information

PRISMA

PRISMA 2020 Checklist

Section and Topic	Item #	Checklist item	Location where item is reported
			Sources and Search Strategy; Appendix
Selection process	8	Specify the methods used to decide whether a study met the inclusion criteria of the review, including how many reviewers screened each record and each report retrieved, whether they worked independently, and if applicable, details of automation tools used in the process.	Materials and Methods → Study Selection and Data Extraction; Results → Study Selection; Figure 1
Data collection process	9	Specify the methods used to collect data from reports, including how many reviewers collected data from each report, whether they worked independently, any processes for obtaining or confirming data from study investigators, and if applicable, details of automation tools used in the process.	Materials and Methods → Study Selection and Data Extraction; Statistical Analysis
Data items	10a	List and define all outcomes for which data were sought. Specify whether all results that were compatible with each outcome domain in each study were sought (e.g. for all measures, time points, analyses), and if not, the methods used to decide which results to collect.	Materials and Methods → Statistical Analysis; Results →

PRISMA

PRISMA 2020 Checklist

Section and Topic	Item #	Checklist item	Location where item is reported
			Study Characteristics; Results → Study Outcomes
	10b	List and define all other variables for which data were sought (e.g. participant and intervention characteristics, funding sources). Describe any assumptions made about any missing or unclear information.	Materials and Methods → Study Selection and Data Extraction; Statistical Analysis; Table 1
Study risk of bias assessment	11	Specify the methods used to assess risk of bias in the included studies, including details of the tool(s) used, how many reviewers assessed each study and whether they worked independently, and if applicable, details of automation tools used in the process.	Materials and Methods → Risk of Bias Assessment; Results → Risk of Bias; Figures 2–4
Effect measures	12	Specify for each outcome the effect measure(s) (e.g. risk ratio, mean difference) used in the synthesis or presentation of results.	Materials and Methods → Statistical Analysis

Section and Topic	Item #	Checklist item	Location where item is reported
Synthesis methods	13a	Describe the processes used to decide which studies were eligible for each synthesis (e.g. tabulating the study intervention characteristics and comparing against the planned groups for each synthesis (item #5)).	Materials and Methods → Statistical Analysis; Results → Study Outcomes
	13b	Describe any methods required to prepare the data for presentation or synthesis, such as handling of missing summary statistics, or data conversions.	Materials and Methods → Statistical Analysis
	13c	Describe any methods used to tabulate or visually display results of individual studies and syntheses.	Materials and Methods → Statistical Analysis; Table 1; Figures 5–9
	13d	Describe any methods used to synthesize results and provide a rationale for the choice(s). If meta-analysis was performed, describe the model(s), method(s) to identify the presence and extent of statistical heterogeneity, and software package(s) used.	Materials and Methods → Statistical Analysis
	13e	Describe any methods used to explore possible causes of heterogeneity among study results (e.g. subgroup analysis, meta-regression).	Discussion → Osteopathic Manipulativ

PRISMA 2020 Checklist

Section and Topic	Item #	Checklist item	Location where item is reported
			e Treatment; Discussion → Abdominal Massage; Discussion → Limitations
	13f	Describe any sensitivity analyses conducted to assess robustness of the synthesized results.	Sensitivity analysis was conducted to assess the robustness of the synthesized results where possible; Analysis is presented in Figure 10.
Reporting bias assessment	14	Describe any methods used to assess risk of bias due to missing results in a synthesis (arising from reporting biases).	Limitations
Certainty assessment	15	Describe any methods used to assess certainty (or confidence) in the body of evidence for an outcome.	Certainty of evidence was assessed using the GRADE approach; detailed ratings and justifications are provided in the Appendices

PRISMA 2020 Checklist

Section and Topic	Item #	Checklist item	Location where item is reported
**RESULTS**			
Study selection	16a	Describe the results of the search and selection process, from the number of records identified in the search to the number of studies included in the review, ideally using a flow diagram.	PRISMA flow diagram
	16b	Cite studies that might appear to meet the inclusion criteria, but which were excluded, and explain why they were excluded.	Narrative explanation
Study characteristics	17	Cite each included study and present its characteristics.	Table 1
Risk of bias in studies	18	Present assessments of risk of bias for each included study.	ROB paragraph and Figures 2-4
Results of individual studies	19	For all outcomes, present, for each study: (a) summary statistics for each group (where appropriate) and (b) an effect estimate and its precision (e.g. confidence/credible interval), ideally using structured tables or plots.	Table 1; Figures 2–4; Results (narrative synthesis sections)
Results of syntheses	20a	For each synthesis, briefly summarise the characteristics and risk of bias among contributing studies.	Table 1; Figures 2–4
	20b	Present results of all statistical syntheses conducted. If meta-analysis was done, present for each the summary estimate and its precision (e.g. confidence/credible interval) and measures of statistical heterogeneity. If comparing groups, describe the direction of the effect.	Figures 5–9; Results (Meta-analysis sections)
	20c	Present results of all investigations of possible causes of heterogeneity among study results.	Discussion
	20d	Present results of all sensitivity analyses conducted to assess the robustness of the synthesized results.	Not performed due to limited number of studies and substantial heterogeneity.
Reporting biases	21	Present assessments of risk of bias due to missing results (arising from reporting biases) for each synthesis assessed.	Limitations
Certainty of evidence	22	Present assessments of certainty (or confidence) in the body of evidence for each outcome assessed.	Not performed
**DISCUSSION**			
Discussion	23a	Provide a general interpretation of the results in the context of other evidence.	Discussion
	23b	Discuss any limitations of the evidence included in the review.	Discussion-

PRISMA

PRISMA 2020 Checklist

Section and Topic	Item #	Checklist item	Location where item is reported
			Limitations
	23c	Discuss any limitations of the review processes used.	Discussion-Limitations
	23d	Discuss implications of the results for practice, policy, and future research.	Discussion/ Conclusion
**OTHER INFORMATION**			
Registration and protocol	24a	Provide registration information for the review, including register name and registration number, or state that the review was not registered.	Not prospectively registered
	24b	Indicate where the review protocol can be accessed, or state that a protocol was not prepared.	Protocol not prepared
	24c	Describe and explain any amendments to information provided at registration or in the protocol.	Not applicable
Support	25	Describe sources of financial or non-financial support for the review, and the role of the funders or sponsors in the review.	No external funding
Competing interests	26	Declare any competing interests of review authors.	Declared no interests
Availability of data, code and other materials	27	Report which of the following are publicly available and where they can be found: template data collection forms; data extracted from included studies; data used for all analyses; analytic code; any other materials used in the review.	Included in Supplementary

From: Page MJ, McKenzie JE, Bossuyt PM, Boutron I, Hoffmann TC, Mulrow CD, et al. The PRISMA 2020 statement: an updated guideline for reporting systematic reviews. BMJ 2021;372:n71. doi: 10.1136/bmj.n71.
This work is licensed under CC BY 4.0. To view a copy of this license, visit https://creativecommons.org/licenses/by/4.0/

Postoperative bowel dysfunction, including delayed gastrointestinal recovery and postoperative ileus, is a common complication that increases morbidity and prolongs hospitalization. This systematic review and meta-analysis evaluated the effects of manual medicine interventions, including osteopathic manipulative treatment (OMT) and abdominal massage, on postoperative bowel function. Eligible studies evaluating hands-on manual therapies in postoperative patients were identified through searches of MEDLINE/PubMed, Google Scholar, the Cochrane Library, Semantic Scholar, and ClinicalTrials.gov (through March 16th 2026) and assessed for risk of bias using standardized tools. Random-effects meta-analyses were performed using the inverse variance method. Seventeen studies were included. Both OMT and abdominal massage were associated with a statistically significant reduction in time to first bowel movement. OMT was additionally associated with reduced hospital length of stay (mean difference −2.46 days, 95% CI [−4.52, −0.41]). Time to first flatus showed a favorable but non-significant trend, with substantial heterogeneity. Limitations include heterogeneity in study design, small sample sizes, and inclusion of non-randomized studies. Overall, manual medicine appears to be a beneficial, non-pharmacologic adjunct in postoperative care, though further large, multicenter trials are needed.
